# Effects of slit width on water permeation through graphene membrane by molecular dynamics simulations

**DOI:** 10.1038/s41598-017-18688-x

**Published:** 2018-01-10

**Authors:** Taro Yamada, Ryosuke Matsuzaki

**Affiliations:** Tokyo University of Science, 2641 Yamazaki, Noda, Chiba, 278–8510 Japan

## Abstract

Graphene membranes can be used for nanoscale filtration to remove atoms and are expected to be used for separation. To realize high-permeability and high-filtration performance, we must understand the flow configuration in the nanochannels. In this study, we investigated the applicability of continuum-dynamics laws to water flow through a graphene slit. We calculated the permeability of the flow through a slit using classical molecular dynamics (MD) and compared the MD simulation results for different Knudsen numbers (*Kn*) to predictions based on the no-slip model and slip model. Consequently, the flow through the graphene nanoslit was treated as slip flow only in the range of *Kn* < 0.375. This study provides guidelines for the development of graphene filtration membranes.

## Introduction

The research of Geim *et al*.^1^ in 2004 regarding the isolation of one-atom-thick graphene indicated that graphene can be used in its single-layer form, i.e., as a two-dimensional (2D) material. Their discovery was awarded the 2010 Nobel prize in Physics and received significant attention. Because the 2D structure allows electrons to move only in the plane of the material, graphene has excellent characteristics including mechanical strength^2^, chemical stability^3^, thermal conductivity^4^, and electrical conductivity^5^. Recently, improvements in manufacturing techniques (e.g., chemical vapor deposition (CVD)^6^ and ultrasonication^7^) have enabled the inexpensive fabrication of single-layer graphene. Graphene is used not only alone, but also in a wide range of composite materials, such as electrode materials^8^, biomaterials^9^, and filtration materials^10^.

Experimental and simulation studies focusing on the interactions of graphene with fluids have been performed. The interactions of graphene with water attract the most attention. For example, Geim *et al*.^11^ fabricated graphene membranes and evaluated their permeability to water and other liquids. They reported that the graphene membranes were almost completely impermeable to liquids like helium but did not impede the permeation of water, which was 10^10^ times faster than that of the other liquids. Because of differences in permeability, studies have been performed regarding the use of graphene for filtration membranes through which only water can permeate. In molecular dynamics (MD) simulations, Cohen *et al*.^12^ obtained water permeation that was 100 times faster than that for commercial reverse-osmosis (RO) membranes.

From the viewpoint of fluid dynamics, permeation through simple structures has long been studied, and hydrodynamic predictions have agreed well with experiments and discrete analysis at the macroscale^13–15^. However, at the nanoscale, the influence of the channel walls is relatively large, and it has been experimentally confirmed that the traditional continuum model and no-slip boundary conditions fail to provide accurate predictions^16–21^. Likewise, these models are probably inappropriate for predicting the fluid transport through graphene composite membranes; the actual performance of such membranes may differ from the expected performance. Usually, nanoscale flows of liquids and gases can be modeled using the Knudsen number (*Kn*). However, *Kn* only shows an experimental range of configurations; the exact values applicable to each form are not clear.

Thus, we conducted MD numerical simulations of the fluid flow through graphene nanoslit structures to investigate the applicable limitation value of the continuum-dynamics laws. We would like to propose a generalized indicator that can be used for any fluids including water. Although chemical properties are important, the width of the slit is the most important parameter that affects experiment. Therefore, only slit width is controlled in this study. In general, the graphene membranes have nanoporous or stacked structure. The nanoporous structure is formed by removing some atoms from pure graphene and the characteristic length, i.e., the diameter of the hole, changes discretely. Therefore, it is not possible to investigate the influence of the characteristic length in detail near the transition region. The stacked structure has various parameters (e.g., slit width, interlayer distance, and size of one flake), and it is difficult to determine the characteristic length. Therefore, in order to determine the characteristic length from only the slit width and subsequently treat it as a free variable, we study the single-layer slit structure. We calculated the water permeability using the results of the MD simulations and compared them with predictions based on the no-slip and slip models.

## Results

### Hydrodynamic model

**Figure 1:**
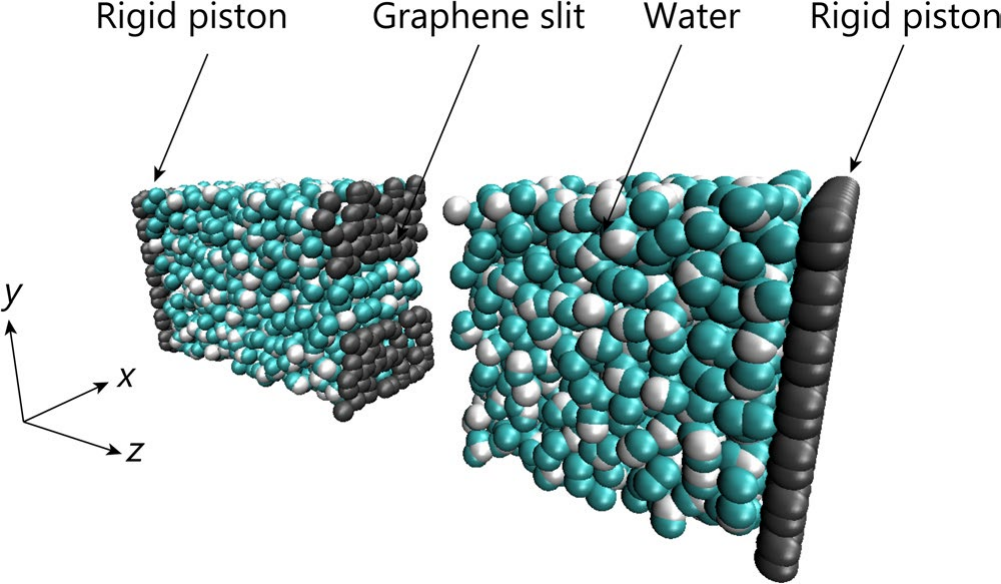
Schematic of computational setup. The graphene slit is located at the center of the unit cell. The rigid pistons located on both sides of *z* axis move only in the +*z* direction at a constant speed of *v*_*p*_. Water molecules are placed on either side of the slit. The mass density of the unit cell is 1 g/cm^3^.

We consider the flow through single-layer graphene slits, as depicted in Fig. 1, where the graphene sheet has a slit of width *d* = 0.464–3.16 nm. The applicability of the continuum law to such nanoscale flows is characterized by the non-dimensional Knudsen number (*Kn*), which is equal to the ratio of the mean free path of fluid molecules *λ* to the characteristic channel dimension *L*_*s*_:

1 $$Kn=\frac{\lambda }{{L}_{s}}\cdot $$

**Figure 2:**
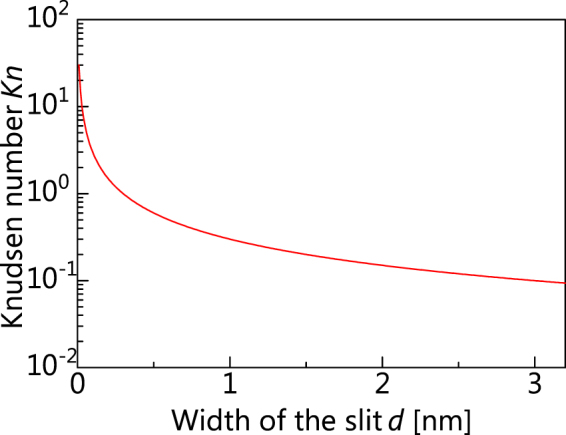
Width of the slit versus Knudsen number. The Knudsen number (*Kn*) can be obtained as *Kn* = *λ*/*L*_*s*_, where *λ* is the mean free path of the water molecules and *L*_*s*_ is the characteristic channel length. Here, *L*_*s*_ is equal to the width of the slit (*d*). Because liquid molecules do not have a mean free path, we used the lattice spacing of water molecules (*δ* = 0.3 nm) instead of *λ*.

Because we consider the flow of liquid water, the lattice spacing for water *δ* (=0.3 nm) is substituted for *λ* ^22^. The characteristic length *L*_*s*_ can be regarded as the slit width *d*, and as shown in Fig. 2, and *Kn* is inversely proportional to *d*. The flow regimes are empirically divided into four (Table 1) according to *Kn* ^23^. We focus on the transition regime (0.464 < *d* < 3.16 nm, i.e. 0.095 < *Kn* < 0.6). In this regime, it is expected that as *d* increases, the flow exhibits a slip tendency, and as *d* decreases, the flow exhibits a free-molecular flow tendency, and the applicability of the slip flow is degraded.

**Table 1:** Knudsen number regimes.

*Kn*	Flow condition	Continuum dynamics
*Kn* < 10^−3^	Continuum flow	Applicable
10^−3^ < *Kn* < 10^−1^	Slip flow	Applicable
10^−1^ < *Kn* < 10	Transition regime	Not applicable
10 < *Kn*	Free-molecular flow	Not applicable

Permeability is an indicator that expresses the ease with which fluid can pass through a membrane. As the permeability increases, filtration requires less energy. The water permeability through the membrane *K* is obtained as follows:

2 $$K=\frac{Q}{{\rm{\Delta }}P},$$

where *Q* is the flux through the membrane, and Δ*P* is the transmembrane pressure drop. In the calculations using the MD simulation results, we consider the flow through slits with width *d*. Assuming that the 2D channel follows the theoretical model of permeability, note that flux *Q* is a per unit length in the *x* direction(see Fig. 1).

By solving the continuum-dynamics equations, the theoretical permeance for the no-slip model *K*_*non-slip*_ and the slip model *K*_*slip*_ is written as follows^24^:

3 $${K}_{non-slip}=\frac{\pi {d}^{2}}{32\mu },$$

4 $${K}_{slip}=\frac{\pi {d}^{2}}{32\mu }+\alpha d,$$

where the viscosity of water *μ* is taken form ref.^25^. As with the density profile, the viscosity of water in nanoscale show a spatial distribution. Equations (3) and (4) were derived by assuming constant viscosity. In many cases, this assumption is applicable on the macro scale. We focus on the difference between macroscale flow and nanoscale flow. Therefore, for the macroscale flow equations (3) and (4), we assume that it is natural to consider the viscosity to be a constant. We define a membrane system using a characteristic number that is equal to the slip velocity *u*_*s*_ at the solid-liquid interface divided by the pressure drop Δ*P*. *α* is a proportionality constant with a linear relationship between *u*_*s*_ and Δ*P* assuming.

### MD results

**Figure 3:**
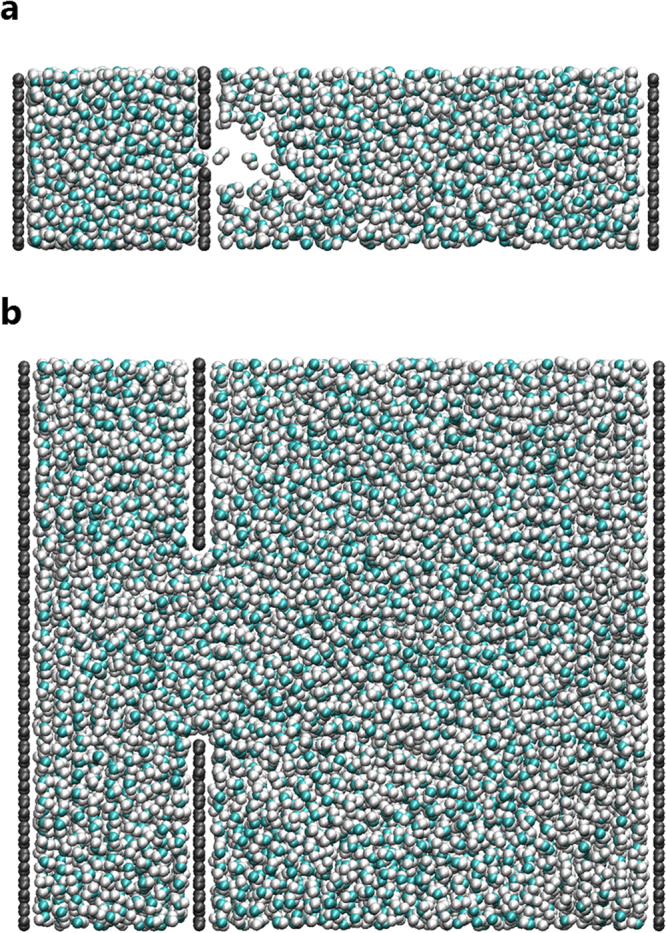
Snapshots of flows through slit in graphene membrane. (**a**) Slit width of *d* = 0.464 nm (*Kn* = 0.65). (**b**) Slit width of *d* = 3.16 nm (*Kn* = 0.094).

Snapshots of our flow simulations with slit widths of *d* = 0.464 and 3.16 nm (*Kn* = 0.65 and 0.095, respectively) and a piston velocity of *v*_*p*_ = 0.0001 nm/fs are shown in Fig. 3 (see Supplementary Fig. 1 for snapshots for slits of all widths *d* at the same step). When the slit width is small, i.e., *Kn* = 0.65, only one water molecule can pass at a time, and a low-density region is observed immediately after the passing of each molecule. This phenomenon differs from continuum flow. The cavitation phenomenon occurs due to the low pressure in the area after permeation. When the piston wall moves, the volume of the downstream side increases. When the slit width is narrow, the volume increase due to the water permeated through the membrane is less than the volume increase due to piston movement. Thus, the pressure in the local region after the water passes through the membrane falls, and cavitation occurs. Although the cavitation phenomenon itself is caused by the movement of the piston wall in the downstream direction, it is important to emphasize that the membrane permeation flow rate is smaller than the flow rate created by the piston wall. It indicates that the density of the fluid changes, violating incompressibility condition assumed when deriving equations (3) and (4).

On the other hand, this tendency is not observed for *Kn* = 0.094, i.e., a wide slit, indicating that the flow tendency changes with in the measurement range (0.094 < *Kn* < 0.65). According to the transition regime defined in Table 1, the flow form changes from a slip flow to a free-molecular flow as *d* decreases.

**Figure 4:**
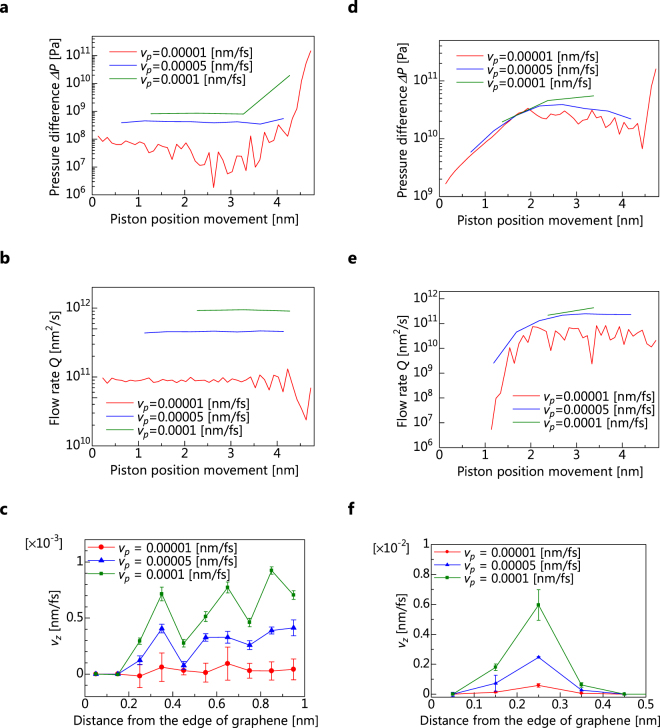
Time distribution and velocity profiles. (**a**,**d**) Pressure difference Δ*P* as a function of the piston position movement from the initial positions at a piston velocity of *v*_*p*_ for *d* = 3.16 nm (**a**) and *d* = 0.464 nm (**d**). (**b**,**e**) Flow rate *Q* as a function of *v*_*p*_. The time-averaged *Q* values were calculated using the average value for the same range as Δ*P* for *d* = 3.16 nm (**b**) and *d* = 0.464 nm (**e**). (**c**,**f**) Velocity profiles of water molecules passing through graphene slits for *d* = 3.16 nm (**c**) and *d* = 0.464 nm (**f**). The error bars indicate that the standard error for the piston position movement from the initial positions is in the of ~1–3.5 nm.

**Figure 5:**
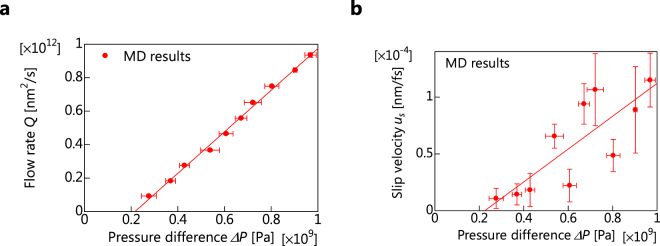
Flow rate and slip velocity. (**a**) Linear relationship between the flow rate *Q* and the pressure difference Δ*P* for a slit width of *d* = 3.16 nm (*Kn* = 0.094). (**b**) Linear relationship between the slip velocity *u*_*s*_ and the pressure difference Δ*P* at a slit width of *d* = 3.16 nm (*Kn* = 0.094). The error bars represent the standard error of each *v*_*p*_ with the same initial conditions.

The time distributions for Δ*P* and *Q* for *d* = 3.16 nm (*Kn* = 0.094) are shown in Fig. 4(a,b), respectively. When *V*_p_ is small, the pressure difference is about 5 × 10^7^ Pa, which is about the same as the standard deviation of the pressure difference obtained from the relaxation (=2 × 10^7^ Pa). Therefore, the pressure difference vibrates more sharply when *V*_p_ is smaller, because variation due to time evolution is larger than that due to the flow. When the distance between the membrane and the piston is smaller than approximately 4 nm, rapid changes of Δ*P* and *Q* are observed. Therefore, we use the average values of Δ*P* and *Q* the when piston-position movements are in the range of 1.0–3.5 nm, and the variation is small in this regime. The relationship between Δ*P* and *Q* at *d* = 3.16 nm (*Kn* = 0.094) is shown in Fig. 5(a). The correlation coefficient for Δ*P* and *Q* is 0.997, and there is a strong linear relationship between them. However, the permeability for the same *d* in equation (2) is not determined uniquely, because the y-axis intercept of the correlation line is not 0. Hence, we consider the inclination of the approximate straight line as the value of the permeability.

Figure 4(c) shows velocity profile between slits at *d* = 3.16 nm. In any *v*_*p*_, the water molecules approximately 0.3 nm from the end of the slit move faster than those in the peripheral part. Thus, it appears that a layer of water molecules is formed approximately 0.3 nm from the edge. Accordingly, we consider the average velocity within a distance of 0.3 nm from the edge of the slit as *u*_*s*_. The relationship between Δ*P* and *u*_*s*_ at *d* = 3.16 nm is shown in Fig. 5(b). The correlation coefficient for Δ*P* and *u*_*s*_ is 0.9617, indicating a strong linear relationship, as with Δ*P* and *u*_*s*_. Therefore, we define *α* as the slope of *u*_*s*_ and Δ*P*.

**Figure 6:**
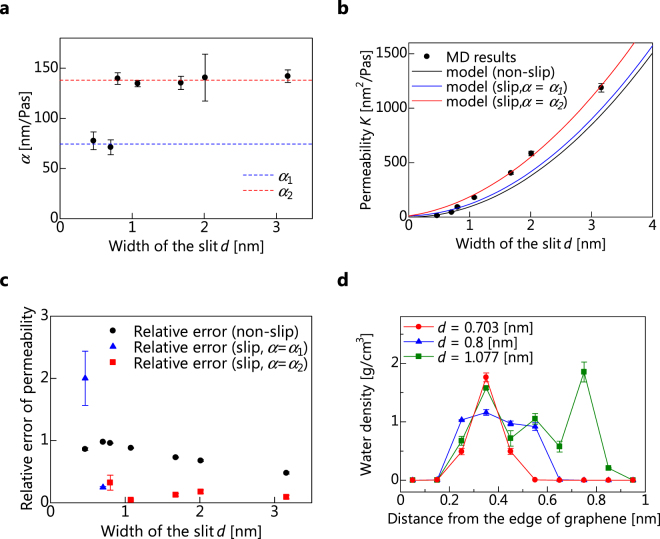
Effect of slit width *d*. (**a**) *α*, which is the slip velocity at the solid–liquid interface divided by the pressure drop, as a function of the slit width. (**b**) Permeability as a function of the slit width. (**c**) Relative error of the permeability as a function of the slit width. (**d**) Density profile of water molecules at *v*_*p*_ = 0.00009 nm/fs. The error bars indicate that the standard error for the piston position movement from the initial positions ranges from approximately 1 nm to 3.5 nm.

Figure 6(a) shows the relationship between *d* and *α*. The tendency of *α* changes at *d* = 0.8 nm (*Kn* = 0.375), and in all regions, *α* does not change because of changes in *d*. We refer to the mean value of *α* at *d* < 0.8 nm as *α*_1_ and that at d > 0.8 nm as *α*_2_. *α*_2_ (=145 nm/Pas) is approximately twice as large as *α*_1_ (=77 nm/Pas). Therefore, the change of *α* at *d* = 0.8 nm is caused not by error but by the changing flow tendency from a slip flow to a free-molecular flow. Thus, we consider that the flow form is a slip flow in the range of *Kn* < 0.375 and a free-molecular flow in the range of *Kn* > 0.375.

The relationship between *d* and *K*, together with the theoretical model of equations (3) and (6), is shown in Fig. 6(b). Compared with the previous results^26^, the permeability that they calculated for *d* = 0.6 nm is only 6 nm2/Pas lower than the second-order approximation of the value we calculated for *d* = 0.464, 0.703, 0.8 nm. Considering that our water molecule model differs from the one used to obtain the previous results, it can be concluded that there is no difference between the two. This fact indicates the validity of our research. For the *d* used for theoretical model calculation, considering the region where water molecules can not exist owing to the repulsive forces between carbon and oxygen atoms, we subtract the van der Waals diameter of a carbon atom (=0.33997 nm) from the distance between the carbon atoms at the end of the slits. The MD simulation results agree better with the theoretical model of the slip condition (*α* = *α*_2_) than with that of the non-slip condition for *d* > 0.8 nm (*Kn* < 0.375). This shows that in the range of *Kn* < 0.375, the flow; is not a non-slip flow but a slip flow, and the continuum-dynamics laws are applicable. It appears that the MD simulation results are close to the non-slip condition theoretical model or the slip-condition theoretical model (*α* = *α*_1_); however, the relative error between the theoretical models and the measurements is shown in Fig. 6(c). The mean value of the relative error for the slip-condition model (*α* = *α*_2_) at *Kn* < 0.375 is 0.15, that for the non-slip model at *Kn* > 0.375 is 0.92, and that for the slip model (*α* = *α*_1_) at *Kn* > 0.375 is 1.12. Compared with the models for *Kn* < 0.375, those for *Kn* > 0.375 have a large error and deviate significantly from the MD results. Consequently, the slip-condition theoretical model (*α* = *α*_1_) and the non-slip condition theoretical model are not applicable in the range of *Kn* > 0.375, and we suggest that the continuum-dynamics laws are also inapplicable in this range.

Figure 6(d) represents the density profile of water molecules passing through slits of various widths—*d* = 0.703, 0.8, and 1.077 nm (*Kn* = 0.43, 0.375, and 0.28, respectively)—at *v*_*p*_ = 0.00005 nm/fs. There is a high-density peak (*ρ* > 1.5 g/cm^3^) for *d* = 0.703 nm (0.35 nm from the edge), and there are two peaks for *d* = 1.077 nm (0.35 and 0.75 nm from the edge), but there are no peaks for *d* = 0.8 nm. Therefore, we consider that the threshold for whether water molecules permeate in a single layer or in multiple layers is approximately *d* = 0.8 nm.

## Discussion

To investigate the applicability of the continuum-dynamics laws, we calculated the permeability of the flow through a graphene slit using MD. Our findings are as follows.

1. As shown in Fig. 6(a), the tendency of *α*—the slip velocity *u*_*s*_ at the solid–liquid interface divided by the pressure difference *ΔP*—changes at *Kn* = 0.375, and *α* does not change according to the slit width *d* in any region.
2. As shown in Fig. 6(b), a discontinuous flow occurs at *Kn* = 0.65. Considering discussion (1), the flow form changes from a slip flow to a free-molecular flow; when *Kn* exceeds 0.375.
3. As shown in Fig. 6(c), the MD simulation results agree well with the slip-condition theoretical model and slip condition is applicable at *Kn* < 0.375. However, the slip-condition model is not applicable at *Kn* > 0.375, and we suggest that the continuum-dynamics laws are not applicable in this range.

When the slit width is small (*d* = 0.464 nm), the flow rate is very low at the beginning. This phenomenon shows that a threshold value of the pressure difference exists for permeation of water. Although the piston moves at a constant speed, water molecules hardly pass through the slit and therefore, the differences in density and pressure between two sides of the slit gradually increase. When the pressure difference reaches a critical value of Δ*P* (in this case, approximately 3.0 × 10^10^ Pa), water molecules suddenly begin to pass through the slit. This sudden pass pushes out the water molecules in the local region just after the slit and a low-density region is formed there. Afterwards, the pressure difference remains nearly constant and no low-density region can be found.

Subtracting the van der Waals diameter of a carbon atom (=0.33997 nm) from *Kn* = 0.375 (*d* = 0.8 nm), according to the previous discussion, gives 0.45 nm. This is the intermediate length between the width of one water molecule (*Kn* = 1.0, i.e., *d* = 0.3 nm) and the width of two water molecules (*Kn* = 0.5, i.e., *d* = 0.6 nm). Similar findings are obtained from the density profile shown in Fig. 6(d). Therefore, we consider that flow changes from a continuum (slip) flow to free-molecular flow when the permeation of water molecules between the slits changes from multiple-layer to single-layer.

On the other hand, the present model does not reproduce an actual phenomenon. In the model, the graphene slits are fixed at a hypothetical point to prevent them from being displaced by the water, but in practice, they are fixed mainly by the covalent bonding of functional groups^27,28^. Consequently, if we design graphene filtration membranes at *Kn* = 0.375, it is unclear whether they will function as intended. However, the system represents a 2D theoretical model, and with regard to the slit width, this study provides useful guidelines for the development of graphene membranes.

## Methods

We calculated the permeability using the Large-scale Atomic/Molecular Massively Parallel Simulator (LAMMPS)^29^, which is an open source MD package. The parameters were taken from the AMBER94 force field^30^, which is often used for biomolecular simulations. The detailed parameters are shown in Supplementary Table 1. In this force field, non-covalent interactions were expressed by the van der Waals term and the electrostatic term, i.e., the sum of the Lennard–Jones (LJ) potential and the Coulomb potential. The cut–off distance for all the LJ potentials was set as 8.0 Å. For long-range Coulombic forces over 8.0 Å, the particle–particle particle–mesh (PPPM) method^31^ was used. The LJ potential parameters for heterogeneous atoms were calculated using the Lorentz–Berthelot combining rules. We used the TIP3P water model^32^, in which a water molecule is treated as a simple rigid body. One of the reasons we use this model is that the AMBER94 force field has been created using the TIP3P water model as a solvent, and adjusted so that the energy, density, and radial distribution function of the water at normal pressure and temperature agree with experimental values^30^. The SHAKE algorithm^33^ was used to constrain the bonds and angles of the oxygen and hydrogen in the water molecules and prevent high-frequency vibrations, which require a short time step. The system dimensions were fixed at (*L*_*x*_, *L*_*y*_, *L*_*z*_) = (18.45, 3*d*, 100.0) Å, and *L*_*y*_ was changed according to the slit width. Equations (3) and (4) assume an infinite length in the *z* direction. However, we set a finite length *L*_z_ in the *z* direction. This approximation can be used because the effect of truncating the far-field flow pattern can be neglected. Actually, the pressure difference and flow rate obtained from the simulation for *d* = 30 Å and *L*_z_ = 200 Å are close to the values for *L*_z_ = 100 Å. Therefore, it is natural to neglect the far-field effect. Periodic boundary conditions were applied in the *x* and *y* directions, and non-periodic boundary conditions were applied in the *z* direction in order for water near the graphene walls not to interact with the periodic cells^34^. When 3*d* was less than twice the cut off distance, *L*_*y*_ was defined to be 3*d* or greater to avoid multiple contributions from the periodic cells. The unit cell contains water molecules at a mass density of *ρ* = 1.0 g/cm^3^. The graphene slits are arranged perpendicularly to the *x*–*y* plane at the position *z* = *L*_*z*_/2 with a slit width of *d*. To prevent the carbon atoms from being displaced by the water, each of the carbon atoms constituting the graphene slits was fixed at a hypothetical point, which only affected the corresponding carbon atom, by covalent bonds with a spring constant of *K*_*r*_ = 100 kcal/(mol∙Å^2^). This imitates the graphene membrane fixed in space with an external force^26^. Since the original graphene film deforms due to the flow pressure, the mechanical properties are not considered in this membrane model. However, the aim is to compare MD results with the results of continuum-dynamics models, in which it is assumed that the membrane does not deform. Moreover, as has been reported previously, the fixture of the graphene membrane does not affect the permeability of the molecules^35,36^. We placed graphene walls at initial atomic coordinates of *z* = 0 and 100.0 Å and moved them at a constant velocity of *v*_*p*_ in the *z* direction as pistons to create a unidirectional flow (Supplementary Movie 1). The motive behind generating flow by a piston is to keep *V*_p_ constant. In equations (3) and (4), it is assumed that the fluid has a constant velocity, *V*_p_, at infinity. If the atoms in a specific region are subjected to a force or acceleration without a piston, the number of atoms in the accelerated region changes; thus, the speed is no longer a constant. Therefore, to satisfy the boundary condition *V*_p_ at infinity, we generate the flow using a piston. If the grand canonical Monte Carlo simulations are performed and the slit width is narrow, the molecules cannot permeate unless the pressure difference does not overcome the threshold. Even if the pistons are used, the molecules cannot also permeate at first. However, since the pressure difference increases as the pistons move, the molecules finally permeate through the slit. Therefore, the aim of using the pistons is to provide the reliable permeation. Ten values of *v*_*p*_ (0.00001, 0.00002, 0.00003, 0.00004, 0.00005, 0.00006, 0.00007, 0.00008, 0.00009, and 0.0001 nm/fs) were calculated to determine the pressure gradients.

All the simulations were performed in the NVT ensemble, and the temperature was maintained at 300 K using a Nosé–Hoover chain thermostat^37^ with a damping parameter of *μ*_*damp*_ = 100 fs. First, the system was relaxed for 1.0 ns using a timestep of 1 fs with the pistons fixed to remove the effect of the initial position. After relaxation, flow simulations were conducted from 0.05 to 0.5 ns by moving the pistons.

The pressure was determined using the virial theorem^38^;

5 $$\begin{array}{c}\langle P\rangle =\frac{N{k}_{B}\langle T\rangle }{V}+\frac{1}{3V}\sum _{i=1}^{N}\sum _{\begin{array}{c}j=1\\ j\ne i\end{array}}^{N\text{'}}\langle {{\bf{r}}}_{ij}\cdot {{\bf{F}}}_{ij}\rangle ,\\ {{\bf{r}}}_{ij}={{\bf{r}}}_{i}-{{\bf{r}}}_{j}\end{array}$$

where *V* is the volume of the system, *T* is the temperature of the system, *k*_*B*_ is the Boltzmann constant, *N* is the number of atoms, *N*′ is the number of atoms included in periodic cells affecting the atom *i*, and **F**_ij_ is the force induced by atom *j* on particle *i*. We denote to the pressures obtained using equation (6) for the local region before and after the water passes through the membrane as *P*_before_ and *P*_after_, respectively, and the difference between them is defined as Δ*P*:

6 $${\rm{\Delta }}P={P}_{before}-{P}_{after}$$

More precisely, the pressure in the region is not uniform, but there are several sharp peaks near the wall, with *P*_before_ and *P*_after_ being the average pressures in the regions including these peaks. These values include the influence of the walls, but can be cancelled by taking the difference between *P*_before_ and *P*_after_ as in equation (6). We calculated flow rate *Q* according to the number of water molecules *dN* that pass per unit time *dt*,

7 $$Q=\frac{M}{\rho {l}_{x}{N}_{A}}\frac{dN}{dt}$$

where *M* is the molecular weight of water, *N*_*A*_ is Avogadro’s constant, *ρ* is the molecular density of water, *l*_x_ is the length of the unit cell in the *x* direction, and *Q* is converted to be the flow rate per unit length in the *x* direction. The error bars on each physical quantity indicate the standard errors obtained from three independent runs, which started with different initial positions of the water molecules.

## Electronic supplementary material

Supplementary information

Supplementary Movie1 (a)

Supplementary Movie1 (b)
