# Supplementary material for: Effects of slit width on water permeation through graphene membrane by molecular dynamics simulations
Source: Sci Rep. 2018 Jan 10;8:339. doi: 10.1038/s41598-017-18688-x (PMC5762883; doi:10.1038/s41598-017-18688-x)
Supplement: Supplementary file 1 — Supplementary information [file 41598_2017_18688_MOESM1_ESM.pdf]

Effects of slit width on water permeation through graphene membrane by molecular dynamics simulations

Taro Yamada[1] , Ryosuke Matsuzaki[1,*]

[1] Tokyo University of Science, 2641 Yamazaki, Noda, Chiba 278-8510, Japan

*Correspondence should be addressed to R.M. (e-mail: rmatsuza@rs.tus.ac.jp)

a

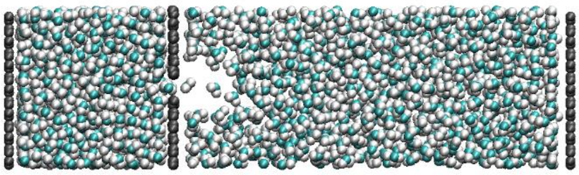

b

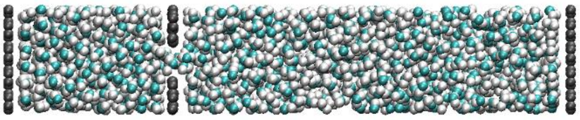

c

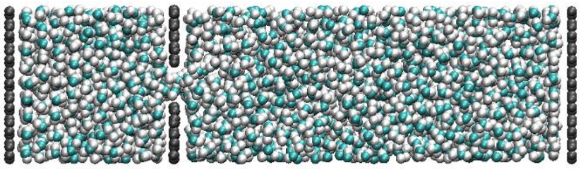

d

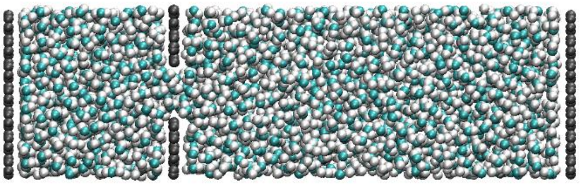

e

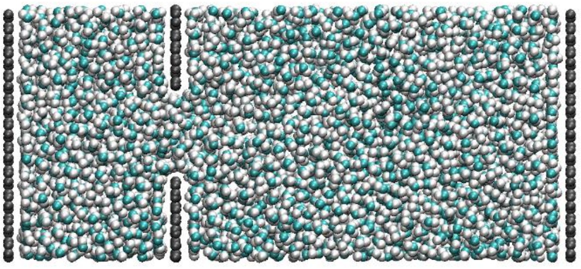

f

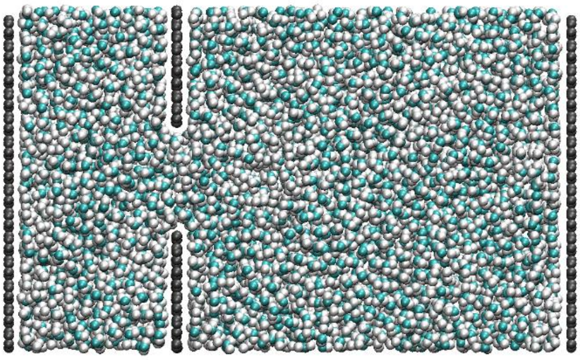

g

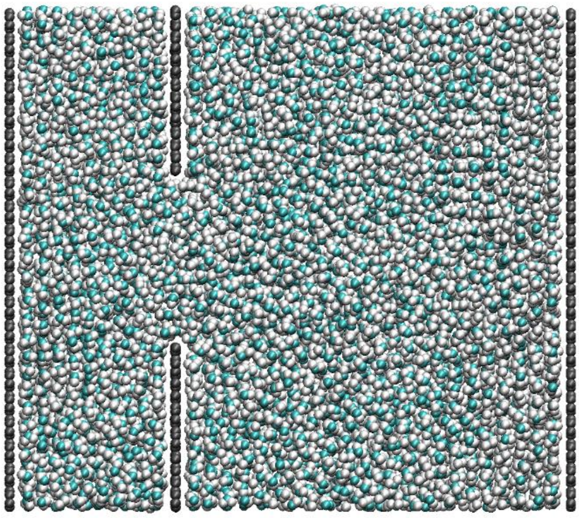

Supplementary Figure 1 | Snapshots of flows through slit in graphene membrane. (**a**) Width of the slit is *d* = 0.464 nm (*Kn* = 0.65). (**b**) Width of the slit is *d* = 0.703 nm (*Kn* = 0.43). (**c**) Width of the slit is *d* = 0.8 nm (*Kn* = 0.375). (**d**) Width of the slit is *d* = 1.077 nm (*Kn* = 0.28). (**e**) Width of the slit is *d* = 1.677 nm (*Kn* = 0.18). (**f**) Width of the slit is *d* = 2.012 nm (*Kn* = 0.15). (**g**) Width of the slit is *d* = 3.16 nm (*Kn* = 0.094).

Supplementary Table 1 | The potential energy-function parameters for the simulations are presented[1]. The C-$C_{virtual}$ bond means that the carbon atoms at the membranes (C) are fixed at frozen atoms ($C_{virtual}$). The atoms of $C_{virtual}$ only affect carbon atoms at the membranes. The interaction parameters between different species in the simulated systems are obtained according to the Lorentz–Berthelot rules.

Atom Parameters				
Atom	ε [kcal/mol]	σ [Å]	mass [g/mol]	q [e]
C	0.0859	3.3997	12.0107	0.0
H	0.0000	0.0000	1.008	+0.52
O	0.1520	3.5366	15.9994	-1.04

Bond Parameters		
Bond	K_r [kcal/(mol·Å^2)]	r_{eq} [Å]
C-C	469.0	1.42
C-$C_{virtual}$	100.0	0.0
O-H	553.0	0.9572

Angle Parameter		
Angle	K_θ [kcal/(mol·radian^2)]	θ_{eq} [deg]
H-O-H	100.0	104.52

Supplementary References

1. Cornell, W. D. *et al.* A second generation force field for the simulation of proteins, nucleic acids, and organic molecules. *Journal of the American Chemical Society* **117**, 5179-5197, (1995).
